# Rare earth contamination of edible vegetation: Ce, La, and summed REE in fungi

**DOI:** 10.1007/s00253-024-13087-5

**Published:** 2024-03-20

**Authors:** Jerzy Falandysz, Anna Kilanowicz, Alwyn R. Fernandes, Ji Zhang

**Affiliations:** 1 Faculty of Pharmacy, Department of Toxicology, Medical University of Lodz, 1 Muszyńskiego Street, 90-151 Łódź, Poland; 2 School of Environmental Sciences, University of East Anglia, Norwich, NR4 7TJ UK; 3 Medicinal Plants Research Institute, Yunnan Academy of Agricultural Sciences, 2238 Beijing Road, Panlong District, Kunming, 650200 China

**Keywords:** Edible fungi, Environment, Health, Metallic elements, Pollution, Soil

## Abstract

**Abstract:**

The increasing and diversified use of rare earth elements (REE) is considered a potential source of pollution of environmental media including soils. This work documents critically overview data on the occurrence of REE in the fruiting bodies of wild and farmed species of edible and medicinal mushrooms, as this was identified as the largest published dataset of REE occurrence in foodstuff. Most of the literature reported occurrences of cerium (Ce) and lanthanum (La), but a number of studies lacked data on all lanthanides. The Ce, La, and summed REE occurrences were assessed through the criteria of environmental geochemistry, analytical chemistry, food toxicology, mushroom systematics, and ecology. Ce and La accumulate similarly in fruiting bodies and are not fractionated during uptake, maintaining the occurrence patterns of their growing substrates. Similarly, there is no credible evidence of variable REE uptake because the evaluated species data show natural, unfractionated patterns in accordance with the Oddo-Harkins’ order of environmental lanthanide occurrence. Thus, lithosphere occurrence patterns of Ce and La as the first and the third most abundant lanthanides are reflected in wild and farmed mushrooms regardless of substrate and show that Ce is around twice more abundant than La. The current state of knowledge provides no evidence that mushroom consumption at these REE occurrence levels poses a health risk either by themselves or when included with other dietary exposure. Macromycetes appear to bio-exclude lanthanides because independently reported bioconcentration factors for different species and collection sites, typically range from < 1 to 0.001. This is reflected in fruiting body concentrations which are four to two orders of magnitude lower than growing substrates.

**Key points:**

•*Original REE occurrence patterns in soils/substrates are reflected in mushrooms*

•*No evidence for the fractionation of REE during uptake by fungi*

•*Mushrooms bio-exclude REE in fruiting bodies*

**Graphical Abstract:**

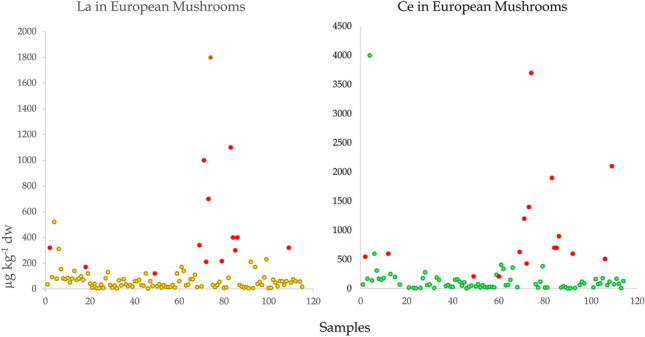

## Introduction

Fungi are an important component of the biosphere, and their production (micro- and macrofungi) is a rapidly growing sector of the food economy worldwide (Royse et al. 2017). This includes developing countries in Africa, Asia, and Central America where locals, indigenous people, or members of the general public forage for both, fruiting bodies and sclerotia (a dense conglomerate of mycelium produced by some species that serves as a food reserve for the fungus) as a food and also as a therapeutic resource. Dozens of species have been successfully farmed, and there are continuous attempts to domesticate new ones (Wasser 2010; Nnorom et al. 2013; Wang et al. 2013; Thawthong et al. 2014; Santiago et al. 2016; Yongabi 2019). Foraging for wild mushrooms is still popular in Europe, e.g., in Italy, Spain, Czechia, Slovakia, Poland, Turkey, Finland, Lithuania, Switzerland, and France, either as a traditional pastime, a seasonal recreation, or for commercial reasons (Læssoe et al. 1996; Isiloglu et al. 2001; Pelkonen et al. 2006; Stijve 2007; Falandysz and Borovička 2013; Kalać, 2016). Globally, 44.2 million tons of mushrooms were produced in 2021 (FAO 2022), but this does not include the quantities of wild mushrooms that are foraged by individuals for personal consumption or for sale, locally. Button mushrooms (champignon), oyster mushrooms, and shitake are still the most popular commercial varieties and dominate the global consumer market for these foods (FAO 2022).

Mushrooms with edible or medicinal value are highly biodiverse, numerous, and have evolved to colonize a variety of soil and plant substrates. Their position, between the plant and animal kingdoms, bestows a meaty texture and taste to the flesh of many edible species, largely resulting from the occurrence and composition of amino acids (Miller et al. 2014; Jaworska et al. 2015; Kalać, 2016). The possibility of combining traditional knowledge of mushrooms in folk medicine (Grzywnowicz 2007; Wasser 2010; Bhatt et al. 2018), with the requirements of modern pharmacy and the search for new bioactive compounds as possible medicines, is currently a challenging field of research (Money 2016; Gründemann et al. 2020). The successful attempts to domesticate some of these have resulted in a diverse range of farmed species—mainly saprotrophs, which can be raised using a wide variety and composition of substrates (often waste agricultural/plant material) (Koutrotsios et al. 2018; Rizzo et al. 2021; Berger et al. 2022). Complementing this use and biodiversity, fungi are now increasingly explored for bioactive organic and inorganic components and as possible agents for the remediation of contaminated land (Sanchez and Demain 2017; Treu and Falandysz 2017).

This work concisely documents and critically overviews literature data on the occurrence of La and Ce in the fruiting bodies of wild and farmed species of edible and medicinal mushrooms. In particular, the evaluation was based on parameters such as environmental geochemistry, cultivation practice, analytical chemistry, food toxicology, mushroom systematics, and ecology. An initial assessment of any potential health risk from REE intake through mushroom consumption was also made.

## Rare-earth elements

Lanthanides, referred to as the rare earth elements (REE; La, Ce, Pr, Nd, Sm, Eu, Gd, Tb, Dy, Ho, Er, Tm, Yb, and Lu), are often reported as light-, medium-, and heavy atomic weight REE. The IUPAC definition also includes Sc and Y, but this inclusion is currently the subject of debate between different scientific disciplines. They are dispersed in soil bedrock, clays, and topsoils and are similarly absorbed from the soil solution by mushrooms and plants, making them amenable to further migration up food webs (Brioschi et al. 2013; Khan et al. 2017; Squadrone et al. 2019; Patel et al. 2023). Within the last few decades, applications of REE in alloys, high-tech materials, and commodities have seen a rapid increase as have the related activities of geological extraction and processing of ores for these metals. REE are increasingly used in modern technologies including magnets in electric motors, metallurgy, the electronics sector, wind turbines, crude oil refining, catalytic converters in the automotive industry, and others (Voncken 2016; Mordor Intelligence 2022). So, in addition to research and investigations into geological resources, production, new applications, inventions, and analytical chemistry, REE also attract growing attention within the environmental, food, and toxicological sciences (Li et al. 2013; Migaszewski and Gałuszka 2016; Doulgeridou et al. 2020; Piarulli et al. 2021; Brouziotis et al. 2022; Falandysz and Fernandes 2023). Typically, REE are present in terrestrial feeds and drinking water at low levels, but the current state of knowledge suggests that they are neither essential nor toxic at current occurrence levels in foods, feeds, and environmental media (Squadrone et al. 2018; Wysocka et al. 2018). From the food toxicology point of view, data on the toxicity of the full range of REE to humans are incomplete, while their occurrence and typical concentrations in plant-based foodstuff are reported as “natural” and without advisories or precautions (Squadrone et al. 2018; Doulgeridou et al. 2020). Recent experiments on toxicity using omics-based approaches (with micro-fungus *Saccharomyces cerevisiae*) have identified some biological functions and pathways that may be disrupted by some medium and heavy atomic weight REE and also the key genes and proteins that are associated with this mode of toxicity (Pallares et al. 2023). These effects were not identified for the light atomic weight members (Ce, La, and Nd), which have analogous features and can be a substitute for calcium (Ca) in some bacteria. REE data reported so far on matrices from marine environments (macro algae—seaweeds, fish seem to show higher concentrations in marine than terrestrial species, on a dry weight basis) (Squadrone et al. 2017, 2019).

Among the REE, La, Ce, and Nd have seen the most applications. Cerium is largely used to manufacture aluminim alloys and also as a fluid cracking catalyst for oil refineries, a catalyst for self-cleaning ovens, in engineered nanomaterials, a polishing powder for liquid crystal and glass display panel surfaces, magnetic memory discs, a chemical oxidizing agent, as a yellow colorant in glass and ceramics, as ferrocerium flints for lighters, and as robust intrinsically hydrophobic coatings for turbine blades. Lanthanum is used in high refractive index and alkali-resistant glass, flint, hydrogen storage, battery-electrodes, camera, and refractive telescope lenses, and as a fluid catalytic cracking catalyst in oil refineries (Voncken 2016).

CeO_2_NP and CeO_2_ are engineered Ce-nanoparticles that are used as model compounds in toxicological studies but have also found agricultural and commercial application, with the potential for nanomedicine (Ma et al. 2023). The pressures of promoting innovation and economy, accompanied by the lack of comprehensive toxicological research on new food additives, raise questions on the risk such as the safety of metal nanoparticles that are used directly in food and taken up orally by humans. Even those that have been used for some time, e.g., TiO_2_, SiO_2_, ZnO, Fe_2_O_3_, turn out to be problematic and even risky (Cheng et al. 2023).

This increasing and diverse use of REE is considered a potential source of pollution of environmental media including soils (Qvarforth et al. 2022). Airborne REE, (e.g., La, which is one of the most used REE in fluid catalytic cracking catalysts and is released from local sources of emission such as production sites or oil refineries), behave like classical heavy metals, undergoing atmospheric and also wastewater diffusion, and finally fallout or sedimentation on surfaces (Kulkarni et al. 2007; Migaszewski and Gałuszka 2015; Censi et al. 2017). A recognized example of environmental pollution by REE is that of gadolinium (Gd). For some years now, Gd-based contrast agents have been administered to patients by intravenous injection in order to improve the clarity of magnetic resonance imaging and magnetic resonance angiography scans, as an aid to diagnosis. The dosed Gd is subsequently excreted through the renal system, although there is debate about the proportion of the dose that is retained and can accumulate in the body, e.g., in the brain (Guo et al. 2018; Kanda 2019; Ibrahim et al. 2023). There are reports that such use of gadolinium chelates which are ultimately disposed off through the sewage system results in the contamination of freshwater, drinking water, and beverages that are produced using these waters (Migaszewski and Gałuszka 2016; Schmidt et al. 2019).

## Metallic elements and mushrooms

Micro- and macrofungi are key components of forest ecosystems. They recycle chemical elements and other nutrients, often symbiotically benefiting plants, but they are also food for a myriad of organisms including large animals (Lepp et al. 1987; Berendes and Steinhauser 2022). In addition to the physiological necessity of assimilating essential nutrients including minerals from their substrates, macromycetes inadvertently also uptake a variety of environmental pollutants, both inorganic elements, as well as a range of anthropogenic organic chemicals. This uptake and assimilation are well recognized, particularly for potentially toxic elements (PTEs), and has been extensively studied (Falandysz and Borovička 2013; Falandysz 2016; Braeuer et al. 2000; Strumińska-Parulska et al. 2021; Falandysz et al. 2022a; Golovko et al. 2022, and many others). Consequently, the contents of some elements such as Hg, Pb, Cd, or radiocaesium in mushrooms are regulated in some countries and regions.

The potential of mushrooms for mycelial uptake (and temporal storage), transfer, and accumulation of a given chemical element or compound in the fruiting body is estimated using the concept of the bioconcentration factor (BCF) (Tyler 1980). The BCF is the concentration ratio (quotient) of fruiting body occurrence relative to that of the substrate. It can be estimated using the absolute (total) element concentration in the fruiting body. For soil substrates, some estimations use absolute (or pseudo-total) concentrations but also other (extractable-labile, mobile, or adsorbed fraction) concentration data (Grawunder and Gube 2018; Lipka et al. 2018). BCF values for La and Ce for both wild and farmed mushrooms show bio-exclusion (i.e., the BCF ratio is < 1) (Aruguete et al. 1998; Grawunder and Gube 2018; Koutrotsios et al. 2018; Vukojević et al. 2019; Zocher et al. 2018; Mędyk and Falandysz 2022).

## Ce, La, and ΣREE in forest soils and wood substrates

### Natural concentrations and distribution patterns

**Fig. 1:**
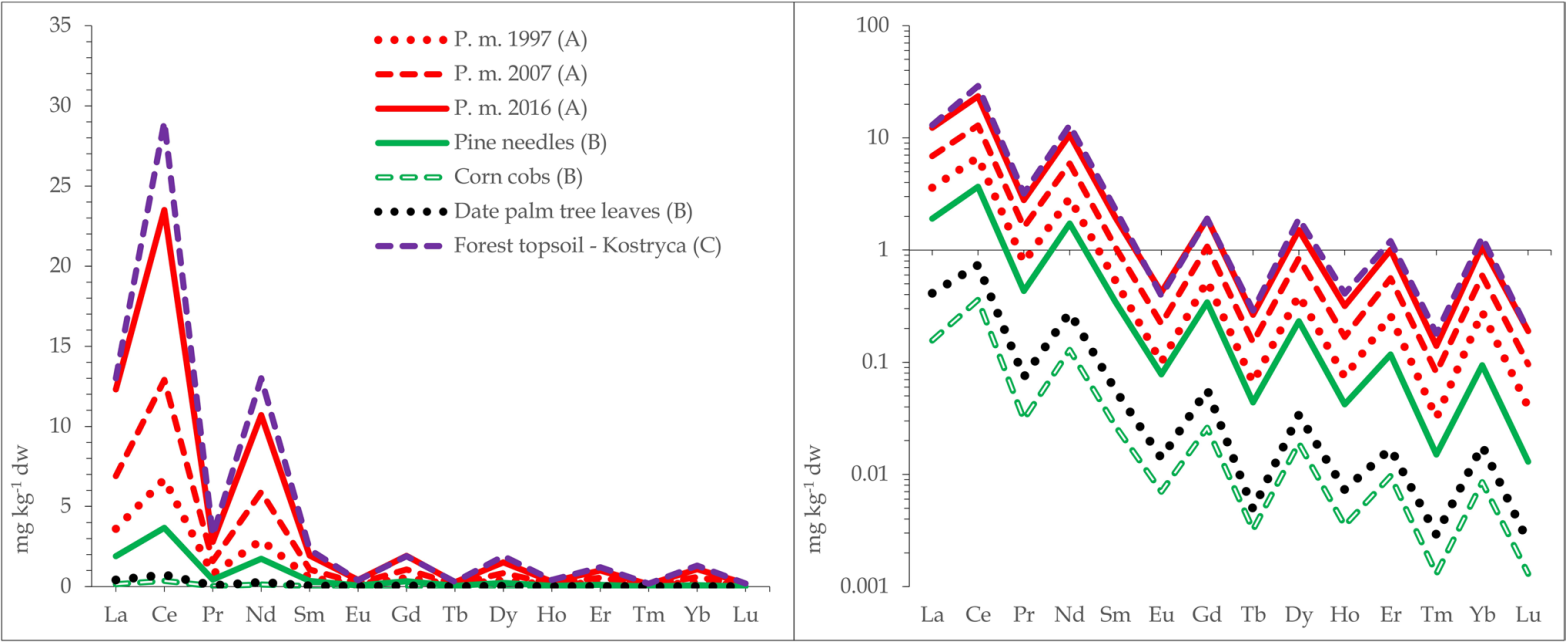
Natural normal- and log-normal distribution pattern of lanthanides in annual rings of pine (*Pinus massoniana*) growing in a REE mining area in China (A), substrates for farmed mushrooms (pine needle, corn combs and data palm tree leaves in Greece; (B) and forest topsoil from Kostryca in Belarus (C) (after Mędyk and Falandysz 2022; Koutrotsios et al. 2018 and Zhang et al. 2019, respectively)

**Fig. 2:**
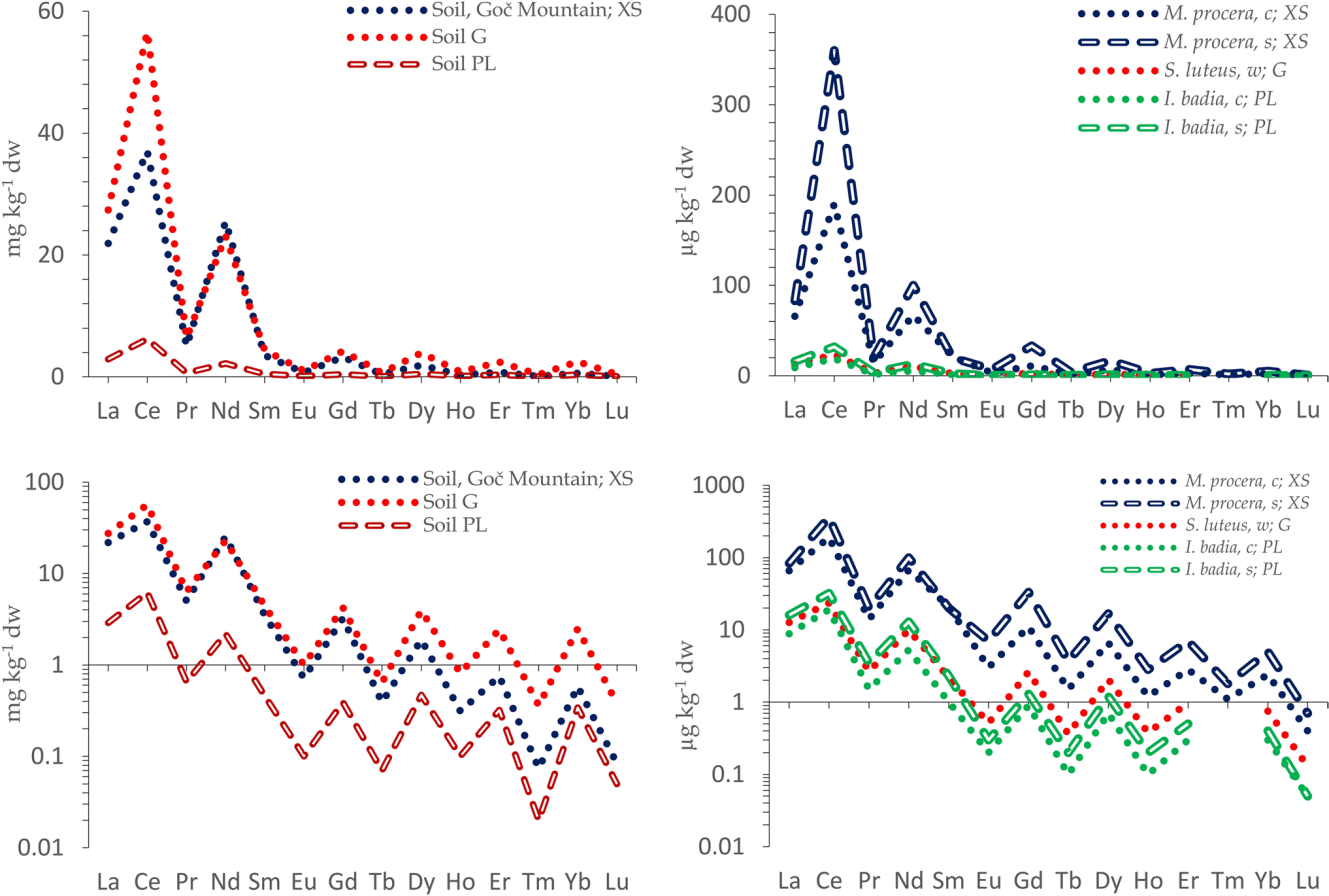
Natural normal- and log-normal distribution pattern of lanthanides in forest topsoils from Serbia (XS), Germany (G), and Poland (PL) and in wild mushrooms (caps, stems, or whole) from Serbia, Germany, and Poland (after Mędyk and Falandysz 2022; Vukojević et al. 2019 and Zocher et al. 2018, respectively)

**Fig. 3:**
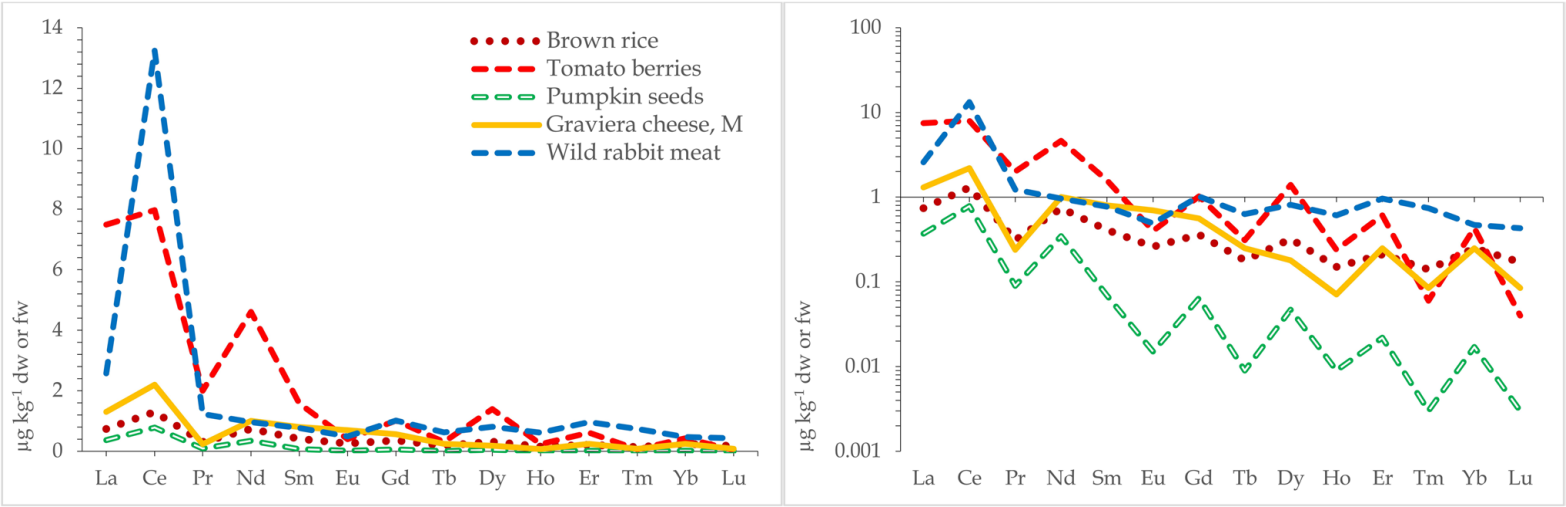
Natural normal- and log-normal distribution pattern of lanthanides in brown rice, tomatoes, pumpkin seeds, cheese, and muscle meat (after Danezis et al. 2017 and 2018; Joebstl et al. 2010; Spalla et al. 2009 and Tagami et al. 2018, respectively)

Cerium was the most abundant REE in forest topsoils that were collected along with mushrooms in Poland/Belarus, Serbia, and Germany contributing 40–44, 33–38, and 42%, respectively to the sum. La was also abundant, contributing 19–21, 22–22, and 20%, respectively contrasting strongly with lutetium, the least abundant REE with contributions of 0.27–0.46, 0.07–0.08, and 0.30%, respectively (Zocher et al. 2018; Vukojević et al. 2019; Mędyk and Falandysz 2022). The occurrence pattern of REE in the soil is reflected in mushrooms, trees, plants, and other food products (a consequence of the Oddo-Harkins’ order of elemental occurrence), thus maintaining the original abundance patterns (Figs. 1, 2, and 3). Absolute concentrations of REE in foods range from low to ultra-low levels, e.g., based on 79, 91, and 65 positive results (that were above the method quantification limit) out of 98 samples of brown rice, dry weight (dw) concentrations of Ce, La, and Lu were 1.3 µg kg^−1^, 0.74 µg kg^−1^, and 0.17 µg kg^−1^ dw, respectively (Fig. 3). Similarly, respective Ce, La, and Lu concentrations were 7.97, 7.49, and 0.04 µg kg^−1^ dw in Italian tomatoes, 2.2, 1.3, and 0.085 µg kg^−1^ fw (fresh weight) in Graviera (Gruyère) cheese from Macedonia, and 13, 2.6, and 0.43 µg kg^−1^ fw in the muscle meat of wild European rabbits *Oryctolagus cuniculus* from Greece (Fig. 3). This preserved occurrence pattern owes much to the similarity of the physico-chemical properties between REE, i.e., they all show similar electronic configurations, ionic radii, and a dominant trivalent oxidation state. Consequently, they also have generally, the same biogeochemical fate—they behave similarly or largely as “one element” as they migrate through food webs (Kabata-Pendias and Pendias 1999). A few REE, e.g., Eu and Ce in part can occur also in other oxidation states in the environment which may sometimes lead to an anomaly in their “shale or chondrite normalized” distribution pattern in environmental media (Migaszewski and Gałuszka 2015; Kwecko 2016). Such an anomaly in the occurrence of Eu in mushrooms has been observed by Borovička et al. (2011) as well as in *Macrolepiota procera* (Falandysz et al. 2017). Similarly, minor anomalies of Eu occurrence were observed for some morphological parts but not for all mushroom samples in another study (Mędyk and Falandysz 2022), or for *Suillus luteus* which showed a positive anomaly for Y (Zocher et al. 2018). Plots of natural normal- and log-normal distribution of REE occurrence in abiotic and biological samples display the characteristic saw-tooth pattern reflecting the mentioned Oddo-Harkins order. This characteristic is very useful for objective (internal or external) assessment and verification of the credibility and analytical quality of a data set. It is therefore important, scientifically justified, and sound to provide the results of determination of a full range of REEs in the tested matrix, instead of only one or a few randomly selected REE, which is sometimes observed in reported data on mushrooms (Table 1).

**Table 1:** La, Ce, and ΣREE in macromycetes (µg kg^−1^ dw; adapted; uncertain or clearly biased data are shown in bold, and with a question mark)

Species	Country/region	Year	*n*	Lanthanum	Median	Range	Cerium	Median	Range	ΣREE	Median	Range	Ref
				Mean ± SD			Mean ± SD			Mean ± SD			
Kingdom: Fungi; Division: Ascomycota; Class: Pezizomycetes													
Order: Pezizales; Family: Morchellaceae; Genus: *Morchella*													
*Morchella elata* Fr													
Whole	Sicily	2014	5(5 ~ 15)	35 ± 28	20	15–79	70 ± 58	40	25–160	170 ± 110	79	51–310	Al19
Whole	Sicily, Monreale	2014	3(3 ~ 9)	***320* ± *150* ?**	***270* ?**	***200***–***490* ?**	***550* ± *260* ?**	***440* ?**	***360***–***840* ?**	***1300* ± *810* ?**	***940* ?**	***790***–***2300* ?**	Al19
*Morchella esculenta* (L.) Pers													
Whole	Sicily	2014	2(2 ~ 6)	92		86—99	170	127	160–190	390	WD	360–420	Al19
Family: Tuberaceae; Genus: *Tuber*													
*Tuber aestivum* Vittad													
Peridium	Germany	2016–2017	27	520 ± 400	WD	WD	4000 ± 5200	WD	WD	8400	WD	WD	Ro19
Gleba	Germany	2016–2017	27	79	WD	WD	140	WD	WD	WD	WD	WD	Ro19
Whole	Germany	2016–2017	27	310 ± 280	WD	WD	600 ± 530	WD	WD	WD	WD	WD	Ro19
Whole	IR, IT, RO, HU, SL	2017–2019	26	155 ± 170	WD	WD	310 ± 380	WD	WD	760	WD	WD	Se20
*Tuber borchii* Vittad													
Whole	ES, IT	2019	4	82 ± 69	WD	WD	170 ± 140	WD	WD	420	WD	WD	Se20
*Tuber magnatum* Picco													
Whole	BG, HR, IT, RO	2018–2019	13	76 ± 46	WD	WD	150 ± 87	WD	WD	380	WD	WD	Se20
*Tuber melanosporum* Vittad													
Whole	ES, FR, IT	2018–2019	8	85 ± 59	WD	WD	180 ± 120	WD	WD	440	WD	WD	Se20
*Tuber indicum* Cooke & Massee													
Whole	China	2018–2019	8	150 ± 140	WD	WD	290 ± 320	WD	WD	720	WD	WD	Se20
Peridium	China	2018–2019	1	910			2100			5100			Se20
Gleba	China	2018–2019	1	34			73			200			Se20
Class: Sordariomycetes													
Order: Hypocreales; Family: Ophiocordycipitaceae; Genus: *Ophiocordyceps*													
*Ophiocordyceps sinensis* (Berk.) G.H.Sung, J.M.Sung, Hywel-Jones & Spatafora													
Cultivated	China	2014–2016	1^x^	***160***	WD	WD	***660* ?**	WD	WD	** > *820* ?**			Ml18
Division: Basidiomycota; Class: Agaricomycetes													
Order: Agaricales; Family: Agaricaceae; Genus: *Agaricus*													
*Agaricus bisporus* (J.E.Lange) Imbach (white strain)													
Cultivated	Switzerland	p.2001		~ 50^La,Ce,Nd^						WD			St01
Cultivated	Poland	2007–2015	72	80 ± 0^(26%)^	WD	< MQL—80	***600* ± *660***^(22%)^ **?**	WD	< MQL – ***1600* ?**	WD	WD	WD	Rz17
Cultivated	Poland	2014–2016	1^x^	140	WD	WD	250	WD	WD	** > *5470* ?**			Ml18
*Agaricus bisporu*s (J.E.Lange) Imbach (brown strain)													
Cultivated	Poland	2007–2015	72	70 ± 10^(33%)^	WD	< MQL—90	< MQL	WD		WD			Rz17
*Agaricus subrufescens* Peck													
Cultivated	Poland	2007–2015	72	80 ± 20^(15%)^	WD	< MQL—110	***200* ± *200***^(44%)^	WD		WD			Rz17
*Agaricus urinascens* (Jul. Schäff. & F.H. Møller) Singer (previous name: *Agaricus arvensis* Schaeff.)													
Wild	Czechia	p.2004	3	97 ± 34	97	63 ± 6–130 ± 11		WD		WD			Řa04
Cultivated	Poland	2010–2015	72	**70 ± 20**^**(52%)**^	WD	< MQL—110	70 ± 20^(33%)^	WD	< MQL—90	WD	WD	WD	Rz17
*Agaricus xanthodermus* Genev													
Whole	Czechia	p.2004	2	***170* ?**		< 30–***320* ?**	WD			WD			Řa04
*Agaricus* sp.													
Whole	China	p.2005	1	***290* ?**			***180* ?**			***1000* ?**			Lu05
Genus: *Calvatia*													
*Calvatia gigantea* (Batsch) Lloyd Previous name: *Langermannia gigantea* (Batsch) Rostk													
Whole	Czechia	p.2004	1	120 ± 11			WD			WD			Řa04
Genus: *Chlorophyllum*													
*Chlorophyllum rhacodes* (Vittad.) Vellinga (previous name: *Macrolepiota rhacodes*)													
Whole	Czechia	p.2004	1	41 ± 5			WD			WD			Řa04
Genus: *Coprinus*													
*Coprinus comatus* (O.F. Müll.) Pers													
Whole	Croatia	2016	1	10			19			70			Iv21
Genus: *Cortinarius*													
*Cortinarius caperatus* (Pers.) Fr. (previous name: *Rozites caperata*)													
Whole	Czechia	p.2004	1	40 ± 10			WD			WD			Řa04
Genus: *Leucoagaricus*													
*Leucoagaricus leucothites* (Vittad.) Wasser													
Whole	Croatia	2016	1	6			13			51			Iv21
Genus: *Lycoperdon*													
*Lycoperdon excipuliforme* (Scop.) Pers									Iv21				
Whole	Croatia	2016	1	5			9			43			
*Lycoperdon perlatum* Pers													
Whole	Czechia	p.2004	2	34		12 ± 3–56 ± 4	WD			WD			Řa04
Whole	Croatia	2016	2	7.5		6–9	13		10–17	55		51–60	Iv21
Genus: *Macrolepiota*													
*Macrolepiota procera* (Scop.) Singer													
Caps	Poland	1994–2004	13 (225)	83 ± 49	71	14–170	180 ± 91	130	30–340	400			Fa17
Whole	Poland	2000–2001	3 (45)	130 ± 58	160	64–170	280 ± 130	340	130–370	610			Fa17
Whole	Poland	2014	1 (3)	30			60			600			Ml16
Whole	Poland	1975–2019	45 (~ 135)^§^	9		< 8–27	70		10–870	514			Si20
Whole	Czechia	p.2004	1	26 ± 7			WD			WD			Řa04
Whole	Croatia	2016	1	5			12			45			Iv21
Caps	Serbia	p.2019	19	66 ± 60		12–220	190 ± 220		30–800	388			Vu19
*M. procera* (Scop.) Singer													
Caps	Serbia	p.2019	12	27 ± 28		7.0–110	150 ± 150		30–700	266			Vu19
Stipes	Serbia	p.2019	19	83 ± 96		5–370	360 ± 30		30–***1700* ?**	661			Vu19
Stipes	Serbia	p.2019	12	140 ± 150		10–490	740 ± 490		130–***1700* ?**	**1152**			Vu19
Family: Amanitaceae; Genus: *Amanita*													
*Amanita citrina* Pers													
Whole	Sweden	2003	4–5	WD		2.1–3.2	WD		4.1–5.2	WD			Ty05
*Amanita flavorubescens* G.F. Atk													
Whole	USA, Illinois	1995–1996	6	WD		260–540	WD		570–1160	WD			Ar98
*Amanita muscaria* (L.) Lam													
Whole	Czechia	p.2004	2	75		< 30–140 ± 10	WD			WD			Řa04
*Amanita pantherina* (DC.) Krombh													
Whole	Japan	1990	1	19			34			WD			Yo97
*Amanita rubescens* Pers													
Whole	Czechia	p.2004	2	36		< 25–60 ± 7	WD			WD			Řa04
Whole	USA, Illinois	1995–1996	10	WD		86–***1769* ?**	WD		210–***2983* ?**	WD			Ar98
Whole	USA, Indiana	1995–1996	WD	WD		< 0.1–200	WD		18–340	WD			Ar98
Family: Hydnangiaceae; Genus: *Laccaria*													
*Laccaria amethystina* (Huds.) Cooke													
Whole	Poland	1994	3 (15)	21 ± 5		15–25	46 ± 12		33–50	160			Fa01
Whole	Poland	2014	1 (4)	30			60			***870* ?**			Ml16
Whole	Poland	2017	2 (180)	18		15–21	37		32–43	83		69–97	Mę22
Family: Lyophyllaceae; Genus: *Lyophyllum*													
*Lyophyllum decastes* (Fr.) Singer (previous name: *Lyophyllum fumosum*)													
Whole	Poland	2014	1 (5)	***60* ?**			30			***860* ?**			Ml16
Genus: *Termitomyces*													
*Termitomyces* sp.													
Cap/stipe	Malaysia	p.1996	1	***3150/3500* ?**			WD			WD			La96
Family: Physalacriaceae; Genus: *Armillaria*													
*Armillariella mellea* (Vahl) P.Kumm													
Whole	Poland	2014	1 (5)	***60* ?**			150			***1440* ?**			Ml16
*Armillariella ostoyae* (Romagn.) Herink													
Caps	Poland	1994	6(60)	70 ± 58		11–180	160 ± 140		22–400	360			Fa01
Genus: *Flammulina*													
*Flammulina velutipes* (Curtis) Singer													
Whole	China	p.2005	1	***140 ?***			***340* ?**			***1200* ?**			Lu05
Whole	Poland	2014	1 (3)	30			***120* ?**			***960* ?**			Ml16
Genus: *Hymenopellis*													
*Hymenopellis radicata* (Relhan) Dörfelt													
Whole	Croatia	2016	1	24			51			144			Iv21
Family: >Marasmiaceae; Genus: *Rhodocollybia*													
*Rhodocollybia butyracea* (Bull.) Lennox (previous name: *Collybia butyracea*)													
Whole	Sweden	2003	4–5	WD		1.9–2.6	WD		3.6–4.9	WD			Ty05
Family: Omphalotaceae; Genus: *Collybiopsis*													
*Collybiopsis peronata* (Bolton) R.H. Petersen (previous name: *Collybia peronata*)													
Whole	Sweden	2003	4–5	WD		6.7–11	WD		13–20	WD			Ty05
Family: Omphalotaceae; Genus: *Collybiopsis*													
*Collybiopsis peronata* (Bolton) R.H. Petersen (previous name: *Collybia peronata*)													
Genus: *Lentinula*													
*Lentinula edodes* (Berk.) Pegler													
Whole	China	p. 2005	2	***350* ?**		***230***–***480* ?**	***270* ?**		***100***–***440* ?**	***1300* ?**		***860***–***1800* ?**	Lu05
Whole	Poland	2014–2016	1^x^	***120* ?**			***110* ?**			** > *2740* ?**			Ml18
Cap/Stipe	Malaysia	p.1996	1	***420/200* ?**			WD			WD			La96
Family: Pleurotaceae; Genus: *Pleurotus*													
*Pleurotus dryinus* (Pers.) P.Kumm													
Whole	Croatia	2016	1	3			6			31			Iv21
*Pleurotus ostreatus* (Jacq. ex Fr.) P.Kumm													
Whole	Poland	2014	1 (5)	***60* ?**			30			***700* ?**			Ml16
Cultivated	Greece	p.2018	7 (28)	23 ± 9	19	15 ± 3–39 ± 9	51 ± 16	49	33 ± 4–75 ± 20	90 ± 27	83	61–130	Ko18
Cultivated	Poland	2014–2016	1^x^	***120* ?**			***210* ?**			** > *5100* ?**			Ml18
Family: Pluteaceae; Genus: *Volvariella*													
*Volvariella volvacea* (Bul.) Singer													
Cultivated	China	2014–2016	1^x^	***170* ?**			***680* ?**			** > *8730* ?**			Ml18
Family: Psathyrellaceae; Genus: *Psathyrella*													
*Psathyrella multipedata* (Peck) A.H.Sm													
Whole	Croatia	2016	1	21			39			146			Iv21
*Psathyrella piluliformis* (Bull.) P.D.Orton													
Whole	Croatia	2016	2	36		19–33	76		35–115	212		110–320	Iv21
Family: Schizophyllaceae; Genus: *Schizophyllum*													
*Schizophyllum commune* Fr													
Whole	Malaysia	p.1996	1	***950* ?**			WD			WD			La96
Family: Strophariaceae; Genus: *Cyclocybe*													
*Cyclocybe cylindracea* (DC.) Vizzini & Angelini (previous name: *Agrocybe cylindracea*)													
Whole	Croatia	2016	1	10			19			64			Iv21
Cultivated	Greece	p.2018	7(28)	29 ± 10	32	13 ± 5–42 ± 2	57 ± 15	57	31 ± 10–81 ± 15	120 ± 40	120	56–170	Ko18
Family: Tricholomataceae; Genus: *Clitocybe*													
*Clitocybe nuda* (Bull.) H.E.Bigelow & A.H.Sm*.* (previous name: *Lepista nuda*)													
Whole	Croatia	2016	1	16			24			85			Iv21
*Clitocybe odora* Bull.) P. Kumm													
Whole	Sweden	2003	4–5	WD		2.8–4.0	WD		4.5–6.7	WD			Ty05
Genus: *Infundibulicybe*													
*Infundibulicybe gibba* (Pers.) Harmaja													
Whole	Croatia	2016	2	13		12–15	22		26–28	90			Iv21
Genus: *Paralepista*													
*Paralepista flaccida* (Sowerby) Vizzini													
Whole	Croatia	2016	1	16			31			94			Iv21
*Paralepista gilva* (Pers.) Raithelh. (previous name: *Lepista gilva*)													
Whole	Poland	2014	1 (6)	***30* ?**			***30* ?**			***930* ?**			Ml16
Genus: *Tricholoma*													
*Tricholoma equestre* (L.) P. Kumm (previous name: *Tricholoma flavovirens*)													
Caps	Poland	1994	3	11 ± 5		7.0–16	21 ± 12		11–35	82			Fa01
Whole	Poland	2014	1 (3)	***120* ?**			***240* ?**			***2150* ?**			Ml16
Whole	Japan	1990	1	***160* ?**			***320* ?**			WD			Yo97
Order: Auriculariales; Family: Auriculariaceae; Genus: *Auricularia*													
*Auricularia auricula-judae* (Bull.) J.Schröt													
Whole	Malaysia	p.1996	1	***2060* ?**			WD			WD			La96
Whole	China	p.2005	1	***330* ?**			***360* ?**			***1470* ?**			Lu05
Whole	Poland	2014	1 (3)	***60* ?**			***210* ?**			***1910* ?**			Ml16
Cultivated	China	2014–2016	1^x^	***370* ?**			***280* ?**			** > *1600* ?**			Ml18
Cultivated	Poland	2014–2016	1^x^	***170* ?**			***410* ?**			** > *2980* ?**			Ml18
*Auricularia nigricans* (Sw.) Birkebak, Looney & Sánchez-Garcia													
Cultivated	China	2014–2016	1^x^	***150* ?**			***640* ?**			** > *6670* ?**			Ml18
Cultivated	Poland	2014–2016	1^x^	***140* ?**			***340* ?**			** > *1620* ?**			Ml18
Family: incerte sedis; Genus: *Pseudohydnum*													
*Pseudohydnum gelatinosum* (Scop.) P.Karst. (previous name: *Tremellodon gelatinosum*)													
Whole	China	p.2010		170			340			> 510			Du10
Order: Boletales; Family: Boletaceae; Genus: *Boletus*													
*Boletus edulis* Bull													
Caps	Poland	1994	4	27 ± 14		16–47	56 ± 25		13–92	140			Fa01
Caps	Poland	1998–2008	5 (72)	34 ± 24	33	14–73	65 ± 46	60	27–140	190 ± 100	190	74–350	Fa22
Stipes	Poland	1998–2008	4 (60)	61 ± 37	56	21–110	120 ± 75	110	40–220	324 ± 170	360	100–**480**	Fa22
Whole	Poland	1998–2008	14 (261)	75 ± 74	51	17–**300**	150 ± 150	95	33–**630**	330 ± 190	310	87–**760**	Fa22
Whole	Poland	1974–2019	44 (~ 132)^§^	75		< 8–***180* ?**	***361* ?**		20–***870* ?**	***1600* ?**			Si20
Whole	Czechia	p.2004	2	110		42–170	WD			WD			Řa04
Whole	Germany	p.2018	1	14			24			63			B18
Whole	Italy	1996	3(~ 1 kg)	***340* ?**		***180***–***490* ?**	***630* ?**		***470**** – ****900* ?**	> ***1610* ?**		> ***890*** > ***2050* ?**	Ma01
*Boletus reticulatus* Schaeff. (previous name: *Boletus aestivalis*)													
Whole	Czechia	p.2004	1	< 20			WD			WD			Řa04
Whole	Sicily	p.2014	1	***1000* ?**			***1200* ?**			** > *2700***			Ve14
*Boletus* spp.													
Whole	Italy (Alps)	1995	~ 1 kg	***210* ± *5* ?**			***430* ± *10* ?**			** > *1000* ?**			Ma01
Whole	China	1995	~ 1 kg	***940* ± *10* ?**			***1880* ± *3* ?**			** > *3860* ?**			Ma01
Genus: *Caloboletus*													
*Caloboletus calopus* (Pers.) Vizzini													
Caps	Yunnan	2015	1 (11)	41			105			227^c^			Mę22
Stipes	Yunnan	2015	1 (11)	60			162			347^c^			Mę22
Genus: *Imleria*													
*Imleria badia* (Fr.) Vizzini (previous name: *Xerocomus badius*, *Boletus castaneus*)													
Whole	Poland	1974–2019	45 (~ 135)^§^	***216* ?**		87–***530* ?**	***384* ?**		100–***700***	***1934* ?**			Si20
Caps	Poland	2007	1 (15)	8.8			19			38^c^			Mę22
Stipes	Poland	2007	1 (15)	16			32			71^c^			Mę22
Caps	Poland	2014	1 (14)	11			19			39			Mę22
Stipes	Poland	2014	1 (14)	41			85			187			Mę22
Stipes	Poland	2017	1 (13)	3.5			7.0			16			Mę22
Whole	Czechia	p.2004	2	85		60 ± 9–110 ± 11	WD			WD			Řa04
Genus: *Hemileccinum*													
*Hemileccinum impolitum* (Fr.) Šutara													
Whole	Sicily		1	***700* ?**			***1400* ?**			** > *2700* ?**			Ve14
Genus: *Leccinellum*													
*Leccinellum lepidum* (H.Bouchet ex Essette) Bresinsky & Manfr.Binder													
Whole	Sicily		2	***1800* ?**		***1000***–***2600* ?**	***3700* ?**		***1400***–***5000* ?**	** > *7100* ?**			Ve14
*Leccinellum pseudoscabrum* (Kallenb.) Mikšík, Previous name: *Leccinum carpini* (R. Schulz) M.M. Moser ex D.A. Reid													
Whole	Czechia	p.2004	1	< 30			WD			WD			Řa04
Genus: *Leccinum*													
*Leccinum scabrum* (Bull.) Gray													
Whole	Poland	1975–2019	45 (~ 135)^§^	14		< 8–53	76		20–310	539			Si20
Whole	Poland	2014	1 (5)	30			20			710			Ml16
Caps	Belarus	2013	1 (15)	55			120			257			Mę22
Stipes	Belarus	2013	1 (15)	86			230			456			Mę22
Genus: *Suillellus*													
*Suillellus queletii* (Schulzer) Vizzini, Simonini & Gelardi													
Whole	Sicily	p.2014	2	***1100* ?**		***500***–***1700* ?**	***1900* ?**		***900 – 3000* ?**	** > *4000* ?**			Ve14
Genus: *Sutorius*													
*Sutorius brunneissimus* (W.F. Chiu) G. Wu & Zhu L. Yang													
Stipes	Yunnan	2015	1 (9)	42			141			273			Mę22
Genus: *Rubroboletus*													
*Rubroboletus lupinus* (Fr.) Costanzo, Gelardi, Simonini & Vizzin													
Whole	Sicily	p.2014	1	***400* ?**			***700* ?**			** > *1400* ?**			Ve14
*Rubroboletus satanas* (Lenz) Kuan Zhao & Zhu L. Yang													
Whole	Sicily	p.2014	1	***300* ?**			***700* ?**			** > *1300* ?**			Ve14
*Rubroboletus rhodoxanthus* (Krombh.) Kuan Zhao & Zhu L.Yang													
Whole	Sicily	p.2014	1	***400* ?**			***900* ?**			** > *1700* ?**			Ve14
Family: Paxillaceae; Genus: *Paxillus*													
*Paxillus involutus* (Batsch) Fr													
Whole	Poland	2014	1 (3)	***30* ?**			***20* ?**			***840* ?**			Ml16
Family: Suillaceae; Genus: *Suillus*													
*Suillus bovinus* (L.) Roussel													
Caps	Poland	1994	3	19 ± 23		5.0–46	42 ± 56		7.0–110	114			Fa01
Whole	Poland	2014	1 (11)	10			20			***730* ?**			Ml16
*Suillus granulatus* (L.) Roussel													
Whole	Japan	1989	1	***100* ?**			***200* ?**			WD			Yo97
*Suillus grevillei* (Klotzsch) Singer													
Caps	Poland	2017	1 (6)	15			2.8			68^c^			Mę22
Stipes	Poland	2017	1 (6)	11			2.0			49^c^			Mę22
*Suillus luteus* (L.) Roussel													
Caps	Poland	1994	3	5.3 ± 2.5		5.0–8.0	9.3 ± 4.7		4.0–13	32			Fa01
Whole	Poland	2014	1 (3)	***210* ?**			***600* ?**			***4850* ?**			Ml16
Whole	Germany	2015	3	6.4 ± 5.4	4.0	2.7–13	11 ± 11	5.1	3.7–23	28 ± 27	15	9.9–59	Zo18
Flesh of caps	Germany	2015	2(8)	4.0		2.3–5.7	7.5		4.0–11	18		10–24	Zo18
Cuticle	Germany	2015	2(7)	11		9.9–13	21		18–24	28		10–45	Zo18
Tubes&spores	Germany	2015	2(7)	4.6		4.0–5.3	8.8		7.8–9.7	21		19–24	Zo18
Stipes	Germany	2015	2(7)	5.9		4.4–7.5	11		8.4–13	26		21–32	Zo18
*Suillus variegatus* (Sw.) Richon & Roze													
Whole	Czechia	p.2004	1	170 ± 11			WD			WD			Řa04
Order: Cantharellales; Family: Cantharellaceae; Genus: *Cantharellus*													
*Cantharellus cibarius* Fr													
Whole	Poland	1998–2008	22 (2562)	39 ± 32	27	1.6–140	62 ± 64	35	< 1.0–250	144 ± 148	77	10–590	Mę23
Whole	Poland	1999–2018	3 (146)	55 ± 35	53	22–91	120 ± 81	100	51–210	260 ± 170	230	110–440	Mę22
Whole	Poland	2014	1 (8)	***30* ?**			***90* ?**			***1050*** ?			Ml16
Whole	Czechia	p.2004	1	90 ± 1			WD			WD			Řa04
*Cantharellus lutescens* (Fr.) Fr													
Whole	Czechia	p.2004	1	230 ± 12			WD			WD			Řa04
*Cantharellus minor* Peck													
Whole	Yunnan	2013	1 (153)	480			940			2072			Mę23
*Cantharellus pallens* Pilát													
Whole	Czechia	p.2004	1	< 7			WD			WD			Řa04
Genus: *Craterellus*													
*Craterellus cornucopioides* (L.) Pers													
Whole	Poland	2016	1 (200)	8.7			22			46			Mę22
Whole	Poland	2017	1 (116)	72			164			346^c^			Mę22
Whole	Croatia	2016	1	50			80			239			Iv21
Family: Hydnaceae; Genus: *Hydnum*													
*Hydnum repandum* L													
Whole	Sweden	2003	4–5	WD		1.6–2.2	WD		2.8–3.6	WD			Ty05
Order: Polyporales; Family: Fomitopsidaceae; Genus: *Fomitopsis*													
*Fomitopsis betulina* (Bull.) B.K.Cui, M.L.Han & Y.C.Dai (previous name: *Piptoporus betulinus*)													
Whole	Poland	2014	1 (4)	***20* ?**			***90* ?**			570			Ml16
Genus: *Laetiporus*													
*Laetiporus sulphureus* (Bull.) Murrill													
Whole	Poland	2014	1 (4)	***60* ?**			***180* ?**			***1640* ?**			Ml16
Family: Ganodermataceae; Genus: *Amauroderma*													
*Amauroderma rude* (Berk.) Torrend													
Whole	China	2014–2016	1^x^	30			50			** > **460			Ml18
Genus: *Ganoderma*													
*Ganoderma applanatum* (Pers.) Pat													
Whole	Poland	2014	1 (4)	60			***510* ?**			***4160* ?**			Ml16
*Ganoderma lucidum* (Curtis) P. Karst													
Whole^xx^	China	p.2009	1	< 5			WD			WD			Xu09
Antler form	China	2014–2016	1^#^	130			***370***			** > *5340* ?**			Ml18
Finger form	China	2014–2016	1^#^	90			***390***			** > *4010* ?**			Ml18
*Ganoderma* spp.													
Whole	Malaysia	p.1996	1	***1650* ?**			WD			WD			La96
Family: Meripilaceae; Genus: *Grifola*													
*Grifola frondosa* (Dicks.) Gray													
Whole	Poland	2014	1 (3)	30			60			***920* ?**			Ml16
Family: Polyporaceae; Genus: *Cerioporus*													
*Cerioporus squamosus* (Huds.) Quélet (previous name: *Polyporus squamosus*)													
Whole	Poland	2014	1 (4)	60			120			***1210* ?**			Ml16
Genus: *Lentinus*													
*Lentinus sajor-caju* (Fr.) Fr. (previous name *Pleurotus sajor-caju*)													
Stipe	Malaysia	p.1996	1	***110* ?**			WD			WD			La96
Genus: *Lignosus*													
*Lignosus rhinoceros* (Cooke) Ryvarden													
Sclerotia	China	2014–2016	1^#^	***200* ?**			***140* ?**			** > *3800* ?**			Ml18
Genus: *Pachyma* (syn. *Wolfiporia*)													
*Pachyma hoelen* (syn. *Wolfiporia cocos* sensu auct.)													
Sclerotia	China	2014–2016	1^#^	***160* ?**			***660* ?**			** > 6*130* ?**			Ml18
Family: Sparassidaceae; Genus: *Sparassis*													
*Sparassis crispa* (Wulfen) Fr													
Cultivated	China	2014–2016	1^#^	***100* ?**			***200* ?**			** > *3750***			Ml18
Order: Russulales; Family: Albatrellaceae; Genus: *Scutiger*													
*Scutiger pes-caprae* (Pers.) Bondartsev & Singer (previous name: *Albatrellus pes-caprae*)													
Whole	Switzerland	1979–1983	3	***320* ± *360* ?**	140	80–***730* ?**	***2100* ± *1700* ?**	***1450* ?**	***860* ~ *4000* ?**	** > *2420* ?**			St01
Whole	Switzerland	1996	1	< 50			74			> 74			St01
Whole	Germany, Bavaria	p. 2001	1	74			170			> 312			St01
Whole	USA, Washington	p. 2001	1	110			**470**			** > *890* ?**			St01
Family: Russulaceae; Genus: *Lactarius*													
*Lactarius blennius* (Fr.) Fr													
Whole	Sweden	2003	4–5	WD		2.6 – 3.2	WD		5.0 – 6.2	WD			Ty05
*Lactarius deterrimus* Gröger													
Whole	Croatia	2016	2	60		28—93	84		31—140	300		150—450	Iv21
*Lactarius hatsudake* Nobuj. Tanaka													
Whole	Japan	1989	1	48			88			WD			Yo97
*Lactarius pubescens* Fr													
Flesh of cap	Germany	2011–2012	23	WD			40 ± 30		9.0—120	WD			Gr18
Flesh of cap	Germany	2011–2012	4	WD			20 ± 20		5.0—60	WD			Gr18
Skin of a cap	Germany	2011–2012	31	WD			800 ± 1200		100—6250	WD			Gr18
Skin of a cap	Germany	2011–2012	16	WD			500 ± 400		100—1500	WD			Gr18
Lamellae	Germany	2011–2012	27	WD			200 ± 300		20–1100	WD			Gr18
Lamellae	Germany	2011–2012	15	WD			80 ± 60		4–220	WD			Gr18
Stipe	Germany	2011–2012	30	WD			80 ± 70		10–330	WD			Gr18
Stipe	Germany	2011–2012	11	WD			30 ± 40		6.0–140	WD			Gr18
*Lactarius rufus* (Scop.) Fr													
Whole	Norway	2014	40	WD	3	< 1–77	< 10	< 10	< 10–200	WD			An18
Genus: *Lactifluus*													
*Lactifluus piperatus* (L.) Roussel (previous name*: Lactarius piperatus*)													
Caps	Poland	2017	1 (8)	57			130			270			Mę22
Stipes	Poland	2017	1 (8)	37			84			190			
Genus: *Russula*													
*Russula mariae* Peck													
Whole	Japan	1990	1	43			90			WD			Yo97
*Russula pectinatoides* Peck													
Whole	USA, Indiana	1995–1996	WD	WD		44–490	WD		81. –1000	WD			Ar98
*Russula virescens* (Schaeff.) Fr													
Whole	Czechia	p.2004	1	< 17			WD			WD			Řa04
Class: Tremellomycetes													
Order: Tremellales; Family: Tremellaceae; Genus: *Tremella*													
*Tremella fuciformis* Berk													
Cultivated	China	2014–2016	1^#^	***60* ?**			***70* ?**			** > *440* ?**			Ml18

*MQL* method quantification limit, 1^x^ (100–130 g dw)

^c^Corrected sum

^§^Sample size for *M. procer*a: 45 (~ 135) [21,909], *B. edulis*: 44 (~ 132) [23,641], *I. badia*: 45 (~ 135) [22,215] and *L. scabrum*: 45 (~ 135) [(22,286]

^xx^Decoct

^#^The samples weigh from 100 to 130 g—possibly dry weight (possibly pooled mushrooms but no information about the quantity of specimens (individuals) in a pool (all examined in 4 replicates—injections or possibly digestions?)

References: Al19 (Alaimo et al. 2019), An18 (Andersson et al. 2018), Ar98 (Aruguete et al. 1998), Ba18 (Bau et al. 2018), Du10 (Du et al. 2010), Fa01 (Falandysz et al. 2001), Fa17 (Falandysz et al. 2017), Fa22 (Falandysz et al. 2022a, b, c, d), Gr18 (Grawunder and Gube 2018), Iv21 (Ivanić et al. 2021), Ko18 (Koutrotsios et al. 2018), La96 (Latiff et al. 1996), Lu05 (Lu et al. 2005), Ma02 (Marzano et al. 2001), Mę22 (Mędyk and Falandysz 2022), Mę23 (Mędyk et al. 2023), Ml16 (Mleczek et al. 2016), Ml18 (Mleczek et al. 2018), Řa04 (Řanda and Kučera 2004), Ro19 (Rossbach et al. 2019), Rz17 (Rzymski et al. 2017), Se20 (Segelke et al. 2020), Si20 (Siwulski et al. 2020), St01 (Stijve et al. 2001), Ty05 (Tyler 2005), Ve14 (Venturella et al. 2014), Vu19 (Vukojević et al. 2019), Xu09 (Xu and Xu 2009), Yo97 (Yoshida and Muramatsu 1997), Zo18 (Zocher et al. 2018)

Biologically, REE are considered to behave similarly to the macroelement, calcium (Ca^2+^) (Liu et al. 2012; Lange and Peiter 2020). In alcohol dehydrogenase of methylotrophic bacteria, REE can take the place of Ca^2+^, particularly in the case of those REE that are more abundant in nature such as La, Ce, Pr, and Nd (Pol et al. 2014; Hibi et al. 2011). In addition to this similarity in behavior to the Ca ion, analogies have also been made with the biological coordination chemistry between REE and Fe^3+^ and Mg^2+^ (Brown et al. 1990; Guo et al. 2016; Cotruvo 2019). Calcium is one of the main macro-minerals and is present in a large excess in relation to the summed REE concentration in foods, including edible mushrooms (Mędyk et al. 2023). On the other hand, Ca also shares behavioral similarities (Saniewski et al. 2016), with barium (Ba) and strontium (Sr), which like Ca are group II alkaline earth metals. Ca, Ba, and Sr significantly exceed the occurrence of REE in mushrooms. For example, in *Suillus grevillei* commonly known as the Larch Bolete or Greville’s bolete, the Ca concentration in caps ranged from 80 to 420 mg kg^−1^ dw, with 220 to 510 mg kg^−1^ dw in the stems (median values for 6 sample sets with 78 specimens), Ba ranged from 0.89 to 7.1 and 1.6 to 7.7 mg kg^−1^ dw, and Sr ranged from 0.31 to 1.8 and 0.79 to 2.3 mg kg^−1^ dw, respectively (Chudzyński and Falandysz 2008). Summed 13–14 REE concentrations in another set of *S. grevillei* were 0.068 mg kg^−1^ dw in the caps and 0.049 mg kg^−1^ dw in the stems (corrected values) (Mędyk and Falandysz 2022). However, possible associations in the occurrence and relationships between REE, Ca, Ba, and Sr in the light of food toxicology are as yet, largely unexplored.

In China, specifically, the REE (largely “a mixture with La, Ce, Nd, and Pr accounting for the main components”) have been used for decades as fertilizers for crops (largely to overcome deficiency symptoms of Ca), and due to overdosing over time, adverse effects have been seen in plants (Redling 2006; Tommasi et al. 2021; He et al. 2022). There is no factual data on whether REE were used in the farming of mushrooms. Farmed mushrooms are largely raised on plant substrates (sawdust, straw, wood, vegetable wastes, etc.), and for some species, additional nutrition can be supplemented through the use of compost like manure (derived from horses and chickens), peat, chalk, and others, but there are no reports of the use of REE (Bhatia et al. 2013; Koutrotsios et al. 2018; Pankavec et al. 2021). REE have been used to promote the growth of livestock but as in the case of crops, it is doubtful if they play any “positive” role (Redling 2006; Schwabe et al. 2012; Tariq et al. 2020).

## Artifacts

### Sampling artifacts—incrusted sand crystals, adhered soil dust, and herbaria samples

Even very minor contamination of mushroom samples with sand (soil dust) debris will result in elevated concentrations of La, Ce, and other REE and also Al, Ca, Co, Cr, Fe, Li, Ni, Sc, Sr, Th, Ti, V, and Y, as has been documented and explained in the literature (Cocchi et al. 2002; Stijve et al. 2001, 2002,  2004). It is difficult to exclude soil dust from fungal samples taken from sandy soil stands and from truffles simply by using dry clean-up methods, and wet clean-up methods, e.g., rinsing with distilled water, can affect water-soluble potassium and phosphates (Stijve et al. 2004). A stipe (stem, stalk) but also a cap (pileus) of some species can, hypothetically, be incrusted with sand (or soil dust), which in practice is impossible to remove completely. Stijve et al. (2004) found artificially high levels of REE and some of the above-listed elements in species such as *Gyrophragmium dunalii* (Fr.) Zeller (current name *Agaricus aridicola* Geml, Geiser, and Royse ex Mateos, J. Morales, J.A. Muñoz, Rey, and C. Tovar (the sand mushroom), *Helvella monachella* (Scop.) Fr., *Morchella dunensis* ((current name *Morchella esculenta* (L.) Pers., (common morel, morel, yellow morel, true morel, morel mushroom, or sponge morel)), *Podaxis pistillaris* (L.) Fr. (the desert shaggy mane), *Psathyrella ammophila* (Durieu and Lév.) P.D. Orton (the dune brittlestem) because of incorporated soil dust. *Gyroporus cyanescens* (Bull.) Quél.) (bluing bolete or cornflower bolete), and other members of the genus *Gyroporus* (*Gyroporaceae*) which grow in sandy soils need particular attention if collected (although most are protected species). Also, the popular *Tricholoma flavovirens* (L.) P. Kumm.—current name *T. equestre* (L.) P. Kumm., (man on horseback or yellow knight), if collected from sandy soil stands would require particular care during cleaning as would many other sand-dwelling species. Mushrooms growing on wood or plant substrates either wild or farmed would generally be free of dust contamination after cutting out the bottom part of the stipe.

Dried mushrooms if bought from retail outlets can be contaminated by soil dust. Karkocha and Młodecki, who studied the nutritive value of dried *Boletus edulis*, *Agaricus bisporus*, *Cantharellus cibarius*, and *Gyromitra esculenta* found that the content of sand in these mushrooms (possibly commercial consignments) ranged from 0.55 to 1.8% (Karkocha and Młodecki 1965). Precaution is advised when working with fungal materials deposited in academic herbaria that can be contaminated with soil substrate residues (Borovička et al. 2011). REE data on mushrooms and their substrates can also be affected by analytical chemistry methodologies that are used for determination.

### Analytical artifacts

Some analytical methodologies and instrumentation that are used in the determination of REE in biological materials can lead to questionable results for REE, including both La and Ce (Table 1). Measurement by X-ray fluorescence analysis (XRF)—a modern non-destructive technique which has been used in the determination of REE in mushrooms has been highlighted as producing unreliable quantitation in part due to the inadequacy of the method quantitation limits (Borovička et al. 2011; Falandysz and Fernandes 2023). Hence, data obtained using XRF were not included in Table 1. A similar non-destructive technique, neutron activation analysis (INAA), allows the determination of many elements in a sample without matrix decomposition, but in the case of REE, INAA has inadequate instrumental and methodological detection and quantification (LOD, LOQ, MDL, MQL) capabilities for a range of REE. Apart from Ce, Nd, and La, other elements with much lower biological occurrence usually fall below the detection or quantitation limits required for mushroom analysis. Using INAA, Řanda and Kučera (2004) were able to provide results only for La (as well as Sc and a few data for Y). Other practical considerations in using INAA are the poor accessibility, high level of technical and safety training, and the high cost of using the technique.

Inductively coupled-emission/optical mass spectroscopy (ICP-A/OES-MS) which has popularly been used for elemental determination is poorly suited for elements at trace or ultra-trace levels including REE in biological materials because of a combination of insufficient instrumental detection limits, background noise, and spectral interferences, particularly during direct analysis of digested sample extracts (Bulska and Ruszczyńska 2017). Due to these limitations, the use of ICP-OES for the determination of REE in mushrooms or other materials (wood, trees, soil, etc.) was found to yield unreliable results (as commented by Zocher et al. 2018; Falandysz 2022a; Falandysz 2022c). Accordingly, only a small subset of the published literature data on La, Ce, and ΣREE in mushrooms using ICP-OES has been included in Table 1, mainly for comparative and illustrative purposes. ICP, coupled to quadrupole mass spectrometry (ICP-MS) with a collision cell, was used in a few studies to determine REE in mushrooms and their substrates. In these studies, the acid-decomposed sample solution was aspirated directly into the plasma (without any further separation from interferences) which resulted in data that were anomalous from the point of view of analytical chemistry and biogeochemistry, i.e., REE results showed random distribution patterns, and many elements had atypically elevated concentrations. So, in common with the ICP-OES data, apart from a few comparative examples, most of these data were not included in Table 1. These issues on the reliability of REE data based on the analytical methodology used have been discussed in more details in other articles (Zocher et al. 2018; Falandysz 2023b; Falandysz and Fernandes 2023; Falandysz et al. 2024).

The credible determination of REE relies on instrumental techniques that are often expensive or use additional purification steps, but other important aspects include the competence and experience of the analytical chemists. For example, sound knowledge of analytical chemistry, geochemistry, environmental and food science, and knowledge of REE occurrence through the literature as well as through the understanding of necessary laboratory infrastructure, which may seem trivial and mundane are still pertinent issues in 2023.

## Patterns—normal and log-normal or shale (or other matrix) normalized and concentration quotients (ratios)

**Fig. 4:**
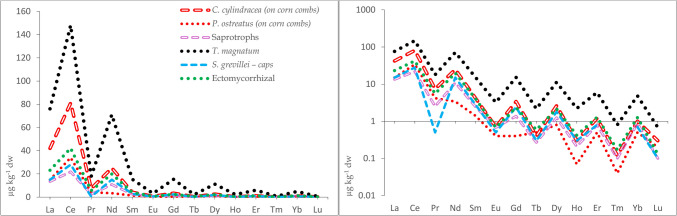
Natural normal- and log-normal distribution pattern of lanthanides in farmed (*C. cylindracea* and *P. ostreatus*) and wild mushrooms; whole saprotrophic and ectomycorrhizal species, caps of *S. grevillei* and in truffle (*T. magnatum*) (after Borovička et al. 2011; Koutrotsios et al. 2018; Mędyk and Falandysz 2022, and Segelke et al. 2020, respectively)

An illustration of the dominance of Ce and La (also Nd) in summed REE occurrence in mushrooms can be observed from the distribution patterns plotted for randomly selected examples in the literature. These are available for farmed and wild epigeous (fruiting above the ground—both ectomycorrhizal and saprotrophic) and hypogeous (below ground) mushrooms, which were obtained using analytical methods with adequate analytical quality and control (AQ/AC) (Fig. 4). Both, relative concentrations and mutual relationships in REE occurrence in mushrooms, their substrates, and some foodstuffs can be explained by the Oddo-Harkins order of elemental occurrence (including REE) as clearly observed by the typical patterns seen in the plotted data in Figs. 1, 2, 3, and 4.

Farmed *Cyclocybe cylindracea* (*C. c.*) and *Pleurotus ostreatus* (*P. o*.) raised on seven plant substrates with natural but different concentrations of La, Ce, and other REE (Koutrotsios et al. 2018) showed BCFs in the ranges: 0.43 to 0.0065 (*C. c.*), 0.56 to 0.0084 (*P. o*.), 1.5 to 0.031 (*C. c*.), and 1.5 to 0.039 (*P. o*.), respectively. The BCF values of Ce calculated for various morphological parts (pileus trama—flesh of a cap, pileipellis—skin of a cap, lamellae—lamella/gills, stipes—stipe/stem) of *Lactarius pubescen*s ranged from 0.014 to 0.0007 (total), 0.22 to 0.011 (mobile), and 2.4 to 0.12 (adsorbed) (Grawunder and Gube 2018). The BCFs of La and Ce available to date for wild and farmed mushrooms (calculated or cited from literature data) and collated in Table 2, generally show bio-exclusion.

**Table 2:** The BCFs of La and Ce available to date, for wild and cultivated mushrooms

Mushroom species	BCF	Ref.*
Wild		
*Cantharellus cibarius*	0.0068, 0.020, 0.0024 (La); 0.0055, 0.023, 0.0027 (Ce)	1
*Craterellus cornucopioides*	0.0080, 0.0010 (La); 0.0086, 0.0012 (Ce)	1
*Imleria badia* (caps, stems)	0.0030, 0.0055, 0.0015, 0.0055, 0.0004 (La); 0.0031, 0.0052, 0.0013, 0.0055, 0.0004 (Ce)	1
*Laccaria amethystina* (whole)	0.0023, 0.0017 (La); 0.0023, 0.0017 (Ce)	1
*Lactifluus piperatus* (caps, stems)	0.0063, 0.0041 (La); 0.0068, 0.0044 (Ce)	1
*Lactarius pubescens*	0.0007, 0.014, 0.0034 and 0.0014 (Ce, total)	2
*L. pubescens*	0.011, 0.22, 0.055 and 0.022 (Ce, mobile fraction)	2
*L. pubescens*	0.12, 2.4, 0.61 and 0.24 (Ce, adsorbed fraction)	2
*Leccinum scabrum* (caps, stems*)*	0.0042, 0.0066 (La); 0.0042, 0.0080 (Ce)	3
*Macrolepiota procera*	0.0041, 0.011 (La); 0.010, 0.038 (Ce)	3
*M. procera* (caps, stems)	0.0030, 0.0020, 0.011, 0.0038 (La); 0.0069, 0.0051, 0.036, 0.0097 (Ce)	3
*Suillus grevillei* (caps, stems)	0.0013, 0.0012 (La); 0.0001, 0.0001 (Ce)	1
*Suillus luteus* (whole)	0.0039 (La); 0.0048 (Ce)	4
Cultivated		
*Cyclocybe cylindracea* (whole)	0.33, 0.27, 0.053, 0.072, 0.43, 0.049, 0.0065 (La); 0.59, 0.12, 0.18, 0.23, 1.5, 0.16, 0.031 (Ce)	5
*Pleurotus ostreatus* (whole)	0.33, 0.27, 0.053, 0.072, 0.43, 0.049, 0.0065 (La); 0.59, 0.12, 0.18, 0.23, 1.5, 0.16, 0.031 (Ce)	5

*Calculated or cited after ^1^Mędyk and Falandysz 2022; ^2^Grawunder and Gube 2018; ^3^Vukojević et al. 2019; ^4^Zocher et al. 2018; ^5^Koutrotsios et al. 2018

These BCFs for Ce and La are in the range of values calculated for Ca in various mushrooms from different collection sites (Jarzyńska et al. 2012; Lipka et al. 2018). Based on the so-called mobile fraction of Ca in the soil substrate which is a portion of the total concentration, BCF values can be greater than 1 (as seen in the above listing for some La and Ce data or for compounds of other elements, largely oxides, which are poorly soluble in pore water). The mycelial network of mushrooms that colonize soil and plant substrates uptake available inorganic compounds readily from the soil solution but also actively search for nutrients originating from rock and mineral bioweathering by excreting chelating agents (Gadd 2017). Mushrooms are much better at bioconcentrating some essential elements like K, Mg, Zn, and Cu (BCF > 1) than La and Ce (and other REE and also Ca, Ba, and Sr) (Jarzyńska et al. 2012; Andersson et al. 2018; Lipka et al. 2018). Toxic elements such as Ag, As, Cd, or Hg occurring at natural concentrations in forest topsoils that are largely similar (Cd) or lower (Ag, As, Hg) than those of Ce or La are much better bioconcentrated (BCF > 1) than REE by mushrooms (Falandysz et al. 2003; Jarzyńska et al. 2012; Árvay et al. 2017; Andersson et al. 2018; Grawunder and Gube 2018; Zhang et al. 2020).

In view of the natural distribution pattern of REE in mushrooms or other biological materials, the values of quotients (concentration ratios) for a given pair of REE should occur within a narrow range regardless of the matrix and type of input data—absolute or shale/chondrite normalized (Falandysz 2023a, 2023c; Falandysz et al. 2024). The La/Ce quotients calculated from available data for certain mushrooms (presented in Table 3) show a narrow range from 0.4 to 0.6, although this may be exceeded in a few examples. Similarly, the La/Ce quotients calculated for shales (Post-Archean Australian Shale, North American Shale Composite, European Shale, and World Shale) that were quoted by Bau et al. were as follows: 0.48–0.51, 0.44–0.47, 0.49–0.51, and 0.49, respectively (Bau et al. 2018). Forest topsoil (0–10 to 0–15 cm layers) at the sites of mushroom collection from Poland showed a La/Ce quotient range from 0.44 to 0.50; the quotient for those collected in Belarus was 0.45 (Mędyk and Falandysz 2022). In three other studies on European mushrooms and soils (Grawunder and Gube 2018; Zocher et al. 2018; Vukojević et al. 2019), the La/Ce quotients for forest topsoil were 0.47 (Ronneburg soil), 0.47 (Jena soil), 0.47 to 0.49 in forested land in Bremen in Germany, and 0.60 to 0.65 in Serbia. In Japanese forests, La/Ce quotients of 0.50 and 0.71 were reported for the litter, fermentation/humifying, and mineral layers of soils, while the ratios were 0.48 and 0.49–0.37 in Sand-dune Regosol and 0.48–0.38 for Andosol (Yoshida and Muramatsu 1997).

**Table 3:** The La/Ce quotients (ratios) for whole fruiting bodies and their morphological parts of several species

Mushroom species	La/Ce quotient	Ref.*
*Amanita pantherina*	0.56	1
*Armillariella* (*mellea*) *ostoyae*	0.44	2
*Boletus edulis*	0.48; 0.60; 0.55, 0.51, and 0.54	2, 3, 4
*Caloboletus calopus*	0.39 and 0.37	5
*Cantharellus cibarius*	0.53, 0.43 and 0.43; 0.77	5, 6
*Craterellus cornucopioides*	0.39 and 0.44	5
*Imleria badia*	0.46, 0.50, 0.58, 0.48, and 0.50	5
*Laccaria amethystina*	0.46; 0.49, and 0.47	2, 5
*Lactarius hatsudake*	0.55	1
*Lactifluus piperatus*	0.44 and 0.44	5
*Leccinum scabrum*	0.45 and 0.37	5
*Macrolepiota procera*	0.46 and 0.47; 0.35, 0.23, 0.18, and 0.19	7, 8
*Russula mariae*	0.48	1
*Suillus bovinus*	0.45	2
*Suillus granulatus*	0.50	1
*Suillus grevillei*	0.54 and 0.55	5
*Suillus luteus*	0.57; 0.54, 0.58, 0.55, 0.52, 0.55, 0.52, 0.51, 0.58, 0.77, 0.54, and 0.72	2, 9
*Tricholoma (equestre) flavovirens*	0.50 and 0.52	1, 2
*Tuber aestivum*	0.5	10
*Tuber borchii*	0.49	10
*Tuber magnatum*	0.51	10
*Tuber melanosporum*	0.47	10
*Tuber indicum*	0.51, 0.42, and 0.47	10
Ectomycorrhizal spp.	0.55	11
Saprobic spp.	0.61	11
Various 15 species	0.57	12
*Pseudohydnum gelatinosum*	0.50	13

*Calculated after ^1^Yoshida and Muramatsu 1997; ^2^Falandysz et al. 2001; ^3^Bau et al. 2018; ^4^Falandysz et al. 2022c; ^5^Mędyk and Falandysz 2022 ^6^Mędyk et al. 2023; ^7^Falandysz et al. 2017; ^8^Vukojević et al. 2019; ^9^Zocher et al. 2018; ^10^Segelke et al. 2020; ^11^Borovička et al. 2011; ^12^Ivanić et al. 2021; ^13^Du et al. 2010

## La and Ce database for mushrooms

**Fig. 5:**
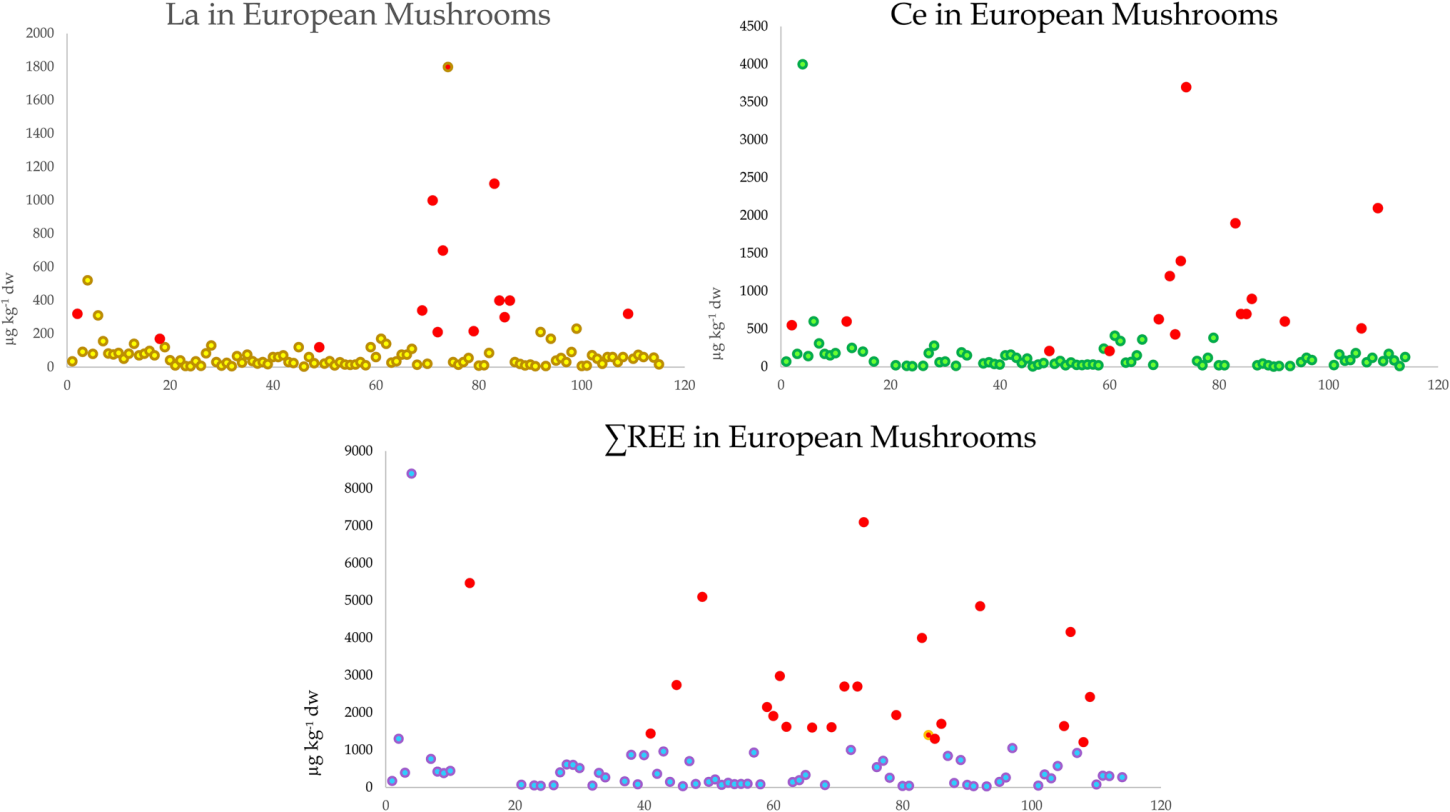
The distribution of La, Ce, and ∑REE in mushroom species (as per Table 1) collected in Europe. Concentrations that appear overestimated are shown in red and originate from ICP-OES and/or some ICP-Quad-MS measurements

An attempt has been made to create a database on the occurrence of La and Ce in mushrooms, both wild and farmed, based on reported literature data. Such a database relies on good-quality data and can be used to establish baseline concentrations for guidance as well as to estimate any future occurrence trends. La and Ce, and summed REE concentration levels, obtained from validated studies (as well as other data with patterns that do not follow the Oddo-Harkins order) were collated (Table 1). Uncertain or clearly biased data as described (Falandysz 2022a, b; 2023a–f; Falandysz et al. 2024) are queried in bold and with a question mark (?). Based on the data in Table 1, the simplified distribution of La and Ce (and summed REE) concentrations in mushroom species collected in Europe is presented in Fig. 5. Concentrations that appear overestimated are shown in red and originate from ICP-OES and/or some ICP-Quad-MS measurements.

Low BCF values and the bio-exclusion of Ce, La, and ∑REE in fruiting bodies suggest that macromycetes are unsuitable for mycomining or the bioremediation of REE, although these occur over a range of concentrations depending on where (above or below the soil or humus substrate), they produce fruiting bodies (Table 1). Two independent studies determined Ce, La, and summed REE in truffles (genus *Tuber*, species *T*. *aestivum* and *T*. *indicum*) that produce subterranean fruiting bodies and reported high concentration in the peridium (a thin outer skin membrane covering the fruitbody) with Ce at 2100–4000 µg kg^−1^ dw and La at 520–910 µg kg^−1^ dw (the quoted uncertainty was very high, i.e. > 100%), while the gleba (the inner fleshy part) concentrations were substantially lower, i.e., Ce at 73–140 µg kg^−1^ dw and La at 34–79 µg kg^−1^ dw (Table 1). The values for gleba were within the range of results reported in wild epigeous fruiting bodies of many species listed in Table 1.

One study reported Ce and La in *Pseudohydnum gelatinosum* (toothed jelly fungus) at concentrations of 340 and 170 µg kg^−1^ dw, respectively, maintaining the biogeochemically sound value of the La/Ce quotient at 0.50 (Table 1). The Ca concentration was 240 mg kg^−1^ dw (Du et al. 2010), which is similar to that cited earlier for *Suillus grevillei*. In order to maintain body structure, the jelly-like plasmodium of the slime mold *Fuligo septica* (*Myxomycetes*, commonly called the dog vomit slime mold or the scrambled egg mold is rich in Ca (8.76% dw, range 4.80 to11.2% dw) chemically close Ba (2550 mg kg^−1^ dw, range 294 to 15,190 mg kg^−1^ dw) and Sr (1290 mg kg^−1^ dw, range 237 to 2190 mg kg^−1^ dw) (Stijve and Andrey 1999). Hypothetically, this very high content of Ca in parallel to Ba and Sr in *F. septica* (and potentially in other jelly fungi with elevated Ca) may imply a potential to accumulate the chemically similar REE, but currently, there are no published studies in support of this hypothesis.

## La and Ce and ∑REE possible intake and risk through mushroom consumption

Precise data on mushroom consumption in different countries is generally unavailable and likely to be an approximation, if available, as this would be based on the production and sale of cultivated species. This is particularly true for areas where foraging of wild mushrooms is widely practiced. Consumption will also vary depending on personal tastes, growing conditions, and the abundance of particular species. Additionally, estimating dietary intake of contaminants or some nutrients from mushroom meals is difficult especially as mushrooms are very rarely eaten raw (fresh, untreated—as a delicacy) as in the case of *Tricholoma matsutake*. Household/culinary (or industrial for both farmed and wild species) processing such as blanching, boiling, blanching, and pickling, typically causes pronounced changes (usually a decrease) in the mineral and trace element concentrations of mushrooms (based on whole-weight, meal weight, or wet weight), depending on the species, the process, and the element (Svoboda et al. 2002; Drewnowska et al. 2017a and 2017b; Falandysz et al. 2019; Pankavec et al. 2019 and 2022). Other cooking methods such as frying, braising, grilling, and sometimes also pickling can lead to a small loss or even a slight concentration increase of an element in a mushroom meal compared to the raw product (on a whole/wet weight basis) (Manzi et al. 2004; Daillant et al. 2013; Falandysz et al. 2022a, 2022b and 2022d; Saba 2021). Further uncertainty in estimating dietary intake arises after consumption because gastrointestinal digestion and bioavailability of metallic elements from a mushroom meal are reported to be limited (Pankavec et al. 2023).

It has been reported that some of the local population in the Yunnan (populated with 46.9 million people in 2022) province of China may consume up to 20–24 kg of wild mushrooms (raw product/fresh weight) per capita, annually (Zhang et al. 2010). An older study suggested that individuals in the UK could consume up to 26 kg (Barnett et al. 1999), which could be considered extreme. However, even if an extreme level of consumption is considered (e.g., 30 kg per individual per annum), the low occurrence levels of Ce and La, and ∑REE in unprocessed mushrooms, combined with the effects of culinary processing and limited gastrointestinal digestion and bioavailability (and thus bioaccessibility), would suggest very limited uptake. Thus, based on the current state of knowledge, there is no evidence of a human health risk through mushroom consumption, or from combined (including other foodstuffs and drinking water) exposure. Clearly, this view could change with new knowledge and insights into the toxicology of these elements, both collectively (the effects of mixtures) and individually, and also if there were concurrent increases in the REE occurrence levels in popular species. Given the expanding use of REE globally, such increases cannot be ruled out, and it would be prudent to initiate surveillance not only in edible mushrooms but also in other foods.

## Conclusions

The occurrences of Ce, La, and summed REE that have been reported in the literature were assessed through the criteria of environmental geochemistry, analytical chemistry, food toxicology, mushroom systematics, and ecology. Based on the type of instrumentation used for measurement and the quality of the analytics used for determination, some data were excluded, particularly when the collective REE occurrence patterns deviated from those predicted by the Odo-Harkins order. The collated data shows that Ce and La accumulate similarly in fruiting bodies and are not fractionated during uptake, maintaining the original occurrence patterns of the substrates in which the fungi grew. There is also no credible evidence that the other REE undergo variable uptake because the evaluated data show natural, unfractionated patterns in accordance with the Oddo-Harkins’ order of environmental lanthanide occurrence. Ce and La were the first and the third most abundant lanthanides in wild and farmed mushrooms regardless of substrate, with Ce occurrence approximately double that of La. The species covered in this report also appear to bio-exclude REE. The fruit body concentrations were two to four orders of magnitude lower than the growing substrates (where reported). This is corroborated by the low values (ranging from 0.001 to below 1) of independently reported bioconcentration factors. There is scant information on the toxicological implications of dietary intake of REE, but the current state of knowledge provides no evidence that wild or cultivated mushrooms pose a health risk either by themselves or when included with the rest of the diet. However, given the growing and varied use of REE in commercial applications, it would be prudent to monitor REE concentrations in environmental and food-related matrices in the future.

## Data Availability

All data generated or analyzed during this study are included in this published article.
